# Knowledge and awareness of iodine intake - survey among Croatian women of reproductive age

**DOI:** 10.11613/BM.2020.010705

**Published:** 2019-12-15

**Authors:** Valentina Vidranski, Anita Radman, Katarina Kajić, Ana Bronić

**Affiliations:** 1 Department for Oncology and Nuclear medicine, Sestre milosrdnice University Hospital Center, Zagreb, Croatia; 2 Department of Clinical Chemistry, Sestre milosrdnice University Hospital Center, Zagreb, Croatia; 3 Laboratory for Medical Biochemistry, General Hospital Bjelovar, Bjelovar, Croatia

**Keywords:** fertility, iodine, knowledge, survey, thyroid diseases

## Abstract

**Introduction:**

Appropriate iodine intake is important for the entire population, especially in fertile women due to decisive role of thyroid hormones in normal foetal brain development. The aim of this study was to investigate knowledge on iodine role among Croatian women of reproductive age.

**Materials and methods:**

The survey was conducted among 378 women of reproductive age during May-September 2018. Data on age, education level, salt intake habits, knowledge of the iodine role and possible presence of thyroid disease were collected and results were presented as numbers or percentage of total number of participants. Comparison between groups was performed by Chi square test.

**Results:**

Of 378 participants, 178 reported to be familiar with the iodine role in the body (P = 0.115). Significantly higher proportion of the younger woman and woman with lower degree of education weren’t familiar with the iodine role (P < 0.001). More woman were introduced to consequences of insufficient than to excessive iodine intake (273 *vs* 213; P < 0.001). In addition, participants mainly weren’t familiar with obligation of salt iodination (P < 0.001). Presence of thyroid disease was recorded in 75 subjects with higher prevalence in subjects 36-49 years (P < 0.001). Presence of thyroid disease was not associated with knowledge on iodine role on health.

**Conclusion:**

Women of reproductive age are not completely aware of the consequences of excessive iodine intake. Thus, further education focusing on more sensitive groups such as woman of younger age should be considered in order to preserve their and their children’s health.

## Introduction

Iodine is an oligoelement essential for the production of thyroxine (T4) and triiodothyronine (T3), hormones that have direct impact on basal metabolism, growth hormone activity as well as on protein, carbohydrate and fat metabolism (*1*). Its metabolism is auto-regulated by the thyroid gland and the effect of thyroid stimulating hormone (TSH) released from the pituitary gland. The availability of iodine in the body depends solely on food intake, and according to the World Health Organisation (WHO), daily needs are between 90 - 120 μg in children, 150 μg for adults and about 250 μg for pregnant and lactating women (*2*). In normal conditions, iodine reserves are sufficient for normal thyroid function for up to 2 months, indicating that the thyroid gland can easily overcome a minor iodine deficiency (*3*).

However, the iodine intake level is considered as “U” shaped, meaning that both low and excessive iodine intake could cause thyroid disorders. The most common disorder due to iodine deficiency is goiter, but in fertile and pregnant women the deficiency could be the cause of decreased fertility, increased incidence of abortion and newborn deaths. Also, it has impact on foetus development and growth disorders during infancy and childhood as well as on cardiovascular and central nervous system disorder such as depression and anxiety in adults (*1*–*5*). The main consequences of excessive iodine intake are hyperthyroidism and autoimmune thyroid disease (*6*).

Iodine saturation is estimated by determination of its concentration in urine as 90% of iodine is excreted through this pathway (*2*, *3*). The cut-off value for the deficiency expressed as median urinary iodine (UI) *per* day in adults is < 100 μg/L whereas concentrations > 300 μg/L are considered excessive. As iodine requirements are greatly increased during pregnancy and lactation, owing to metabolic changes for assessing iodine nutrition, different median UI cut-off values of < 150 μg/L for deficiency and > 500 μg/L for excessive intake are used (*4*).

At the beginning of the 20^th^ century, epidemiological studies showed that iodine deficiency is one of the major public health problems worldwide. Therefore, to prevent disease, salt iodination was initiated as an inexpensive, simple, effective and most common way of nutritive iodine supply (*3*, *7*).

The first research of goiter prevalence in Croatia was conducted in 1953 and as a result, The Croatian Food Law introduced the obligation of salt iodination. In 1996, based on epidemiological research limit of salt iodination was increased from 10 mg on today’s value of 15-23 mg of potassium iodide *per* kilogram of salt. Later studies investigating iodine saturation in Croatia showed that median of UI concentration in 2002 increased to 150 µg/L compared to results obtained in 1997 (< 100 µg/L). The positive trend continued, as in 2015 sufficient iodine saturation among school children was reported by Jukić *et al.* However, knowledge on appropriate iodine intake is important for the entire population, especially for pregnant and breastfeeding women due to decisive role of thyroid hormones in normal brain development during foetal and early postnatal life (*8*, *9*).

So far there is no investigation on knowledge on iodine role in the body and importance of its intake among Croatians. As woman in fertile age are one of most susceptible group the aim of this study was to investigate knowledge on iodine role in the body among women of reproductive age.

## Materials and methods

### Study design

The study was conducted from May to September 2018. The questionnaire with twelve questions, divided into four sections, was created to find out basic data on participants age, level of education, habits on salt intake, knowledge of the iodine role in the body, and possible presence of thyroid disease. To each question answers were provided, except for two questions where positive response was needed and a corresponding explanation should be included. The target population were females of reproductive age, defined by WHO as 15-49 years. The questionnaires in electronic or paper form were distributed to all women in the business environment and friends in Bjelovar, Osijek and Zagreb consenting to participate in the survey. Appropriate sample size was determined by “Sample size calculator” from the data of the Central Bureau of Statistics of 2017, where the number of fertile women in the Republic of Croatia was 930,899 (*10*, *11*). The optimal sample size was 385, with 95% confidence interval (CI) and a 5% error.The questionnaires were distributed to 396 women of reproductive age consenting to participate in survey. Questionnaires without complete or with vague responses were not further considered (N = 18). Thus, 378 surveys were taken in final processing.

### Statistical methods

Data for analysis were obtained by counting. Results were presented as numbers or relative frequencies (percentages) of total number of participants. Comparision between groups was performed by Chi square test. The value of P < 0.05 was considered statistically significant. If result of P < 0.05 were obtained, difference between certain categories was calculated by comparisons of proportions. *Post hoc* tests were used for paired comparisons, applying the Bonferroni correction. Offered answers to question two (“non-qulifield” and “high school degree”), question four (“yes, exclusively” and “yes, equally as sea salt or a stone salt”) and question ten (“monthly” and “every six month”) were, due to statistical proccessing, grouped and obtained results interpreted accordingly. Data analyses were performed using Microsoft Excel 2010 (Microsoft, Redmond, Washington) and MedCalc 11.5.1., statistical software (MedCalc Software, Maraikerke, Belgium).

## Results

Table 1 summarizes the questionnare and frequency of obtained answers from all of 378 survey participants. Of the total number of respondents, 178 (47%) reported to be, whereas 200 (53%) reported not to be familiar with the iodine role in the body (P = 0.115). In general, more respondents were familiar with consequences of insufficient iodine intake compared to excessive iodine intake (273 *vs* 213; P < 0.001).

**Table 1:** Survey results

**Question**	**Response**	**N (%)** **(N = 378)**
1. Your age is:	a) 15-25 years	79 (21)
	b) 26-35 years	158 (42)
	c) 36-49 years	141 (37)
2. Your qualification is:	a) non-qualified	8 (2)
	b) high school degree	152 (40)
	c) bachelor degree	73 (19)
	d) master degree and higher level of qualification	145 (39)
3. Salt intake	a) in larger quantities	67 (18)
	b) moderately	283 (75)
	c) avoid salting	28 (7)
4. Do you consume special types of salt that are not sea or stone salt (*e.g.*, Himalayan, flower, organic-eco salt, *etc.*)	a) yes, exclusively	5 (1)
	b) yes, equally as sea salt or stone salt	29 (8)
	c) yes, sometimes	113 (30)
	d) no, never	231 (61)
5. Do you take iodine in any form of dietary supplements (drops, tablets, capsules)?	a) yes	21 (6)
	b) no	337 (89)
	c) don’t know	20 (5)
6. Do you know that in Croatia there is a The Food Low and The Salt Act, which states that all sea and rock salt must contain 15 to 23 mg iodine *per* kilo salt?	a) yes	145 (38)
	b) no	233 (62)
7. Do you know what the main role of iodine in the body is? If yes, please specify.	a) yes, exact answer was provided	178 (47)
	b) no	200 (53)
8. Insufficient iodine intake*:	a) can lead to osteoporosis and other bone diseases	26 (6)
	b) can cause skin and nail changes, as well as hair loss	54 (13)
	c) can lead to the appearance of drowsiness and deprivation in psychomotor development	243 (57)
	d) does not pose a significant risk to the organism	5 (1)
	e) don’t know	97 (23)
9. Excessive iodine intake:	a) could not lead to undesirable consequences	25 (7)
	b) could cause significant health problems	213 (57)
	c) don’t know	140 (36)
10. How often do you perform determination of thyroid hormones?	a) monthly	8 (2)
	b) every six months	39 (10)
	c) once a year	68 (18)
	d) less than all of the above	263 (70)
11. Do you suffer from any thyroid disease?	a) yes	75 (20)
	b) no	303 (80)
12. On therapy for thyroid disease^†^:	a) yes	50 (67)
	b) no	25 (33)
*The sum of individual responses can vary from 378 since participants could choose more than one answer. ^†^Only respondents who answered “yes” to question 11 should have answered.		

When respondents based on the knowledge of iodine role we considered, a higher proportion of youngest subjects and those with lower degree of education were not familiar with iodine role in the body (19 *vs* 60; P < 0.001, and 58 *vs* 102; P < 0.001, respectively). Also, respondents familiar with iodine role are much more familiar with the fact that salt on the market in Croatia is iodized (109 *vs* 36; P < 0.001) and more acquainted with consequences of insufficient iodine intake compared to its excessive intake (169 *vs* 104, P < 0.001). Salt intake, consumption of specific types of salt and iodine intake in the form of supplements was not different between the studied groups. The difference was not observed for the presence of thyroid disease, althought was recorded that the frequency of annual thyroid hormone determination is higher in subjects that are familiar with iodine role in the body (P = 0.002).

Furthermore, subjects aged between 15 and 25 years, are more familiar with consequences of excessive iodine intake in compare to consequences of insufficient iodine intake (44 *vs* 58; P = 0.006). However, when all subjects were divided based on knowledge of consequences of excessive or insufficient iodine intake any signifficant difference between groups was not recorded (data not shown).

Presence of thyroid disease was recorded in 75 (20%) subjects with higher prevalence in subjects aged in between 36 to 49 years (P < 0.001). Therapy treatment due to thyroid disease was recorded in 50 of 75 subjects. Considering presence of the disease, salt consumption was not different between woman with and without disease, but higher intake of iodine dietary supplements was recorded in woman with thyroid disease (P < 0.001). As expected, the frequency of thyroid hormone determination (monthly and every six month) was significantly higher in woman with thyroid disease (P < 0.001).

## Discussion

Knowledge on appropriate iodine intake is important due to its vital role in the synthesis of thyroid hormones and impact to metabolism. Almost half of participants in our study reported to be familiar with the iodine role in the body. However, results revealed that with its role less familiar were younger woman and those with lower degree of education.

Similar study, conducted by Lucas *et al.*, among pregnant women in Australia reported that knowledge about iodine was generally poor across all areas assessed, but on contrary to our data difference according to age and education has not been found. In addition, they reported poor knowledge among participants on potential outcomes related to iodine deficiency (*12*). Similar results of poor knowledge about iodine intake importance were obtrained in a study on pregnant women living in Northern Ireland (*13*). Combet *et al.* have done a cross-sectional survey among UK-resident women, pregnant or mother to a child (up to 36 months). Awareness about nutritional pregnancy recommendations in general was high (96%) but iodine-specific was very low (12%), as well as the level of confidence of how to achieve good iodine status (28%) (*14*). Garnweidner-Holme *et al.* in the study among pregnant and lactating women in Norway also reported lack of knowledge and awareness about iodine in these population groups. Similar to our study, higher education and increased age was asociated with better knowledge outcomes (*15*). Henjum S. *et al.* made a study on the non-pregnant women, aged 18-30 years, discovering that approximately 40% of the young women have low iodine knowledge score. The questions regarding iodine knowledge in this study demonstrated a low to medium level of awareness about the dietary iodine sources and about the importance of iodine which is similar to results in our study (*16*).

Participants in our study showed that they are better introduced to consequences of insufficient than excessive iodine intake. Despite the fact that, following The Food Law in Croatia, iodine saturation has been improved, it seems that respondents were not familiar with the fact of obligation of salt iodination. In the study performed by Rai *et al.*, women in India at similar age (18-50 years) as in our study with different socioeconomic status and occupations were considered. Results showed lack of knowledge of general terms related to the thyroid gland and its disorders and almost half of the respondents considered hypothyroidism to be treated with iodinated salt (*17*).

In general, it seems that knowledge on appropriate iodine intake is not sufficient and that more effort should be invested in education, especially among fertile age women due to decisive role of thyroid hormones in normal brain development during foetal development. Croatian Institute of Public Health keeps statistics and gives information on thyroid disorders but there is little information on how people get information on importance of proper iodine intake. Thus, further investigations should be performed in order to maintain public health and prevent disease.

According to literature data 1-2% of female population suffer from thyroid disease, while 20% is in subclinical hypothireosis (*18*). Presence of the thyroid disease among participants in our study was relatively high. As expected, it was more frequent among woman aged 36-49 years but was not associated with knowledge on iodine role in the body. However, a worrying fact is that one third of participants with the disease in our study stated not to take any therapy for the disease. Interestingly, study of Rai *et al.* published that over a half of the respondents believe that alternative medicine can treat thyroid disorders (*17*).

Nevertheless, habits of the respondents in our study (salt intake, consumption of special salt types, and iodine intake in the form of dietary supplements) were not significantly different between groups, and were not associated with the presence of thyroid disease. Moreover, taking iodine in the form of dietary supplements among our respondents was very rare, and similar results have been reported by Henjum S. *et al.* in a study of 403, mostly younger subjects, with a frequency of 9% (*16*).

This survey is the first one to assess the knowledge on iodine intake among women in fertile age in Croatia as the most risky group for the occurrence of various disorders due to inappropriate iodine intake. We believe that it could be helpful to other public health projects related to general knowledge on iodine importance and awarenes on thyroid diseases whereas basic demographic data and data on importance of proper iodine intake should be validated against concentration of urine iodine. However, main limitation is that it was performed only on fertile women in three continental Croatian cities Zagreb, Osijek and Bjelovar. Although this cities cover a quarter of Croatian population, future investigations should consider male and female population through a wider area of the country. In order to improve knowledge on iodine intake, further investigation also should collect the data on how and when people get knowledge of iodine intake and thyroid disease prevention. Although epidemiological investigations showed satisfied iodine saturation in Croatia, women of reproductive age are not completely aware of the on consequences of excessive iodine intake. Thus, further education focusing on more sensitive groups such as female at younger age should be considered in order to preserve their and their children health.
